# Polyoxometalate Clusters Confined in Reduced Graphene Oxide Membranes for Effective Ion Sieving and Desalination

**DOI:** 10.1002/advs.202402018

**Published:** 2024-06-17

**Authors:** Yixin Yang, Wan‐Lei Zhao, Yubing Liu, Qin Wang, Ziheng Song, Qinghe Zhuang, Wei Chen, Yu‐Fei Song

**Affiliations:** ^1^ State Key Laboratory of Chemical Resource Engineering Beijing University of Chemical Technology Beijing 100029 P. R. China; ^2^ Quzhou Institute for Innovation in Resource Chemical Engineering Quzhou Zhejiang 324000 P. R. China

**Keywords:** desalination, ion sieving, membranes, polyoxometalates, reduced graphene oxide

## Abstract

Efficient 2D membranes play a critical role in water purification and desalination. However, most 2D membranes, such as graphene oxide (GO) membranes, tend to swell or disintegrate in liquid, making precise ionic sieving a tough challenge. Herein, the fabrication of the polyoxometalate clusters (PW_12_) intercalated reduced graphene oxide (rGO) membrane (rGO‐PW_12_) is reported through a polyoxometalate‐assisted in situ photoreduction strategy. The intercalated PW_12_ result in the interlayer spacing in the sub‐nanometer scale and induce a nanoconfinement effect to repel the ions in various salt solutions. The permeation rate of rGO‐PW_12_ membranes are about two orders of magnitude lower than those through the GO membrane. The confinement of nanochannels also generate the excellent non‐swelling stability of rGO‐PW_12_ membranes in aqueous solutions up to 400 h. Moreover, when applied in forward osmosis, the rGO‐PW_12_ membranes with a thickness of 90 nm not only exhibit a high‐water permeance of up to 0.11790 L m^−2^ h^−1^ bar^−1^ and high NaCl rejection (98.3%), but also reveal an ultrahigh water/salt selectivity of 4740. Such significantly improved ion‐exclusion ability and high‐water flux benefit from the multi‐interactions and nanoconfinement effect between PW_12_ and rGO nanosheets, which afford a well‐interlinked lamellar structure via hydrogen bonding and van der Waals interactions.

## Graphical abstract

A highly efficient polyoxometalate‐assisted in situ photoreduction strategy is applied for the fabrication of the highly ordered rGO‐PW_12_ membranes, which shows excellent water permeance of 0.11790 L m^−2^h^−1^bar^‐1^, high dye rejection of 98.3% for NaCl and ultrahigh water/salt selectivity of 4740. The strong multi‐interactions between PW_12_ and rGO nanosheets contribute to these membranes excellent non‐swelling stability up to 400 h.

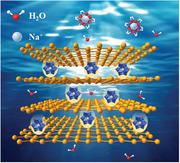

## 1 Introduction

With continuing economic development and increasing demand for water, the importance of clean water resources cannot be overstated.^[^ ^1^ ^]^ Recent studies have demonstrated that membranes based on 2D nanomaterials showed great potential in moderating the global freshwater crisis due to their excellent performance in extracting freshwater from industrial wastewater and saline water.^[^ ^2^ ^]^ The interlayer channels or intralayer pores of 2D membranes in the sub‐nanometer scale was the key step for achieving seawater desalination and water purification, especially the interlayer channels in 1‐nm and sub‐1 nm scales were the dominant transport pathways to achieve precise sieving of most hydrated ions (<1 nm), gas (<0.5 nm) and solvent molecules (<2 nm).^[^ ^3^ ^]^

In this regard, the carbon‐based materials such as graphene oxide (GO) with 2D structures and tunable nanopores or nanochannels attracted tremendous interests.^[^ ^4^ ^]^ The stacked GO was attractive due to its unique water pathway mechanism and the ability to selectively prohibit the undesired ions or gases from passing through.^[^ ^5^ ^]^ Additionally, GO can be obtained from abundant natural graphite by simple methods, which was considered to be cost‐effective.^[^ ^6^ ^]^ However, the GO membranes often suffered from poor stability under practical hydrodynamic flow conditions.^[^ ^7^ ^]^ The swelling of GO membranes in water leaded to expanded interlayer spacing, which significantly decreased the sieving performance during long‐term operation.^[^ ^8^ ^]^ And it was difficult to reduce the interlayer spacing sufficiently to exclude small ions and maintain this spacing when immersed in aqueous solution. Thus, it was challenging to precisely construct stable GO membranes, which can achieve the high rejection of ions while enabling rapid water permeation through the interlayer channels in the filtration process.

In order to develop advanced GO membranes for water purification and desalination, several strategies have been proposed to inhibit the GO membranes’ swelling such as physical confinement,^[^ ^9^ ^]^ chemical decoration,^[^ ^10^ ^]^ complexation,^[^ ^11^ ^]^ small molecule crosslinking,^[^ ^12^ ^]^ and the reduction of GO.^[^ ^13^ ^]^ Among them, the reduction of GO was typically employed to enhance the stability of GO sheets in aqueous media.^[^ ^14^ ^]^ Unfortunately, after reducing GO membranes, the water permeance of the stacked reduced graphene oxide (rGO) membranes was deficient due to the strong capillary force and uncontrollable decrease of the interlayer spacing.^[^ ^15^ ^]^ To overcome the decreased permeability caused by reduction, various nanomaterials were considered to intercalate into rGO such as metal‐organic frameworks (MOFs)^[^ ^13^ ^]^ and multi‐walled carbon nanotubes (MWCNTs).^[^ ^16^ ^]^ However, most reported nanospacers were >1 nm, which was unfavorable for precise sieving at the sub‐nanometer scale. Moreover, the interfacial defects between rGO and nanospacers were difficult to avoid. Therefore, to achieve sieving at the sub‐nanometer scale and to maximize permeability and rejection, suitable nanospacers (<1 nm) were strongly desired to regulate the interlayer spacing of rGO membranes.

Polyoxometalates (POMs) clusters were a group of metal oxides, such as V, Mo, and W, which possessed well‐defined chemical composition and molecular structures.^[^ ^17^ ^]^ H_3_PW_12_O_40_ • xH_2_O (denoted as PW_12_) was a nanosized well‐defined POM with a rigid spherical structure and a uniform size of 1 nm, which made it a suitable nanospacer to precisely regulate the interlayer spacing of membranes. Insertion of PW_12_ clusters with good solubility into 2D rGO laminates can result in the ordered 2D channel^[^ ^18^ ^]^ which can avoid the ubiquitous structure defects brought by disordered packing mode of GO nanosheets with common nanospacers. For example, Song et al. proposed that the construction of POMs‐intercalated layered double hydroxides (LDHs) membranes can precisely control the interlayer spacing and hydrophilicity, promoting spontaneous electron migration and interfacial carrier separation.^[^ ^19^ ^]^ Similarly, the intercalation of PW_12_ into rGO nanosheets was considered to have great potential to construct efficient 2D membranes for tuning and controlling the interlayer spacing to suppress swelling and enhance rejection of ions without sacrificing the water permeance.

In this manuscript, we reported the fabrication of the rGO‐PW_12_ membranes with sub‐nanometer interlayer spacing by using POM‐assisted in situ photoreduction strategy, which exhibited both high water permeance and ion rejections. This new design approach exhibited the following advantages: 1) The intercalation of PW_12_ clusters to the flexible rGO laminates resulted in an ordered 2D lamellar structures; 2) the van der Waals interactions and hydrogen bonding between PW_12_ and rGO nanosheets helped to inhibit the swelling of rGO nanosheets and enhance the stability of the membranes; 3) the interlayer spacing of rGO‐PW_12_ membranes can be controlled in sub‐nanometer range, that was, the size exclusion effect and confinement effect contributed to the efficient ion sieving. Systematic investigation of the ion permeation process by the rGO‐PW_12_ membranes were carried out.

## 2 Results and Discussion

### 2.1 Fabrication of the rGO‐PW_12_ Membranes

**Figure 1:**
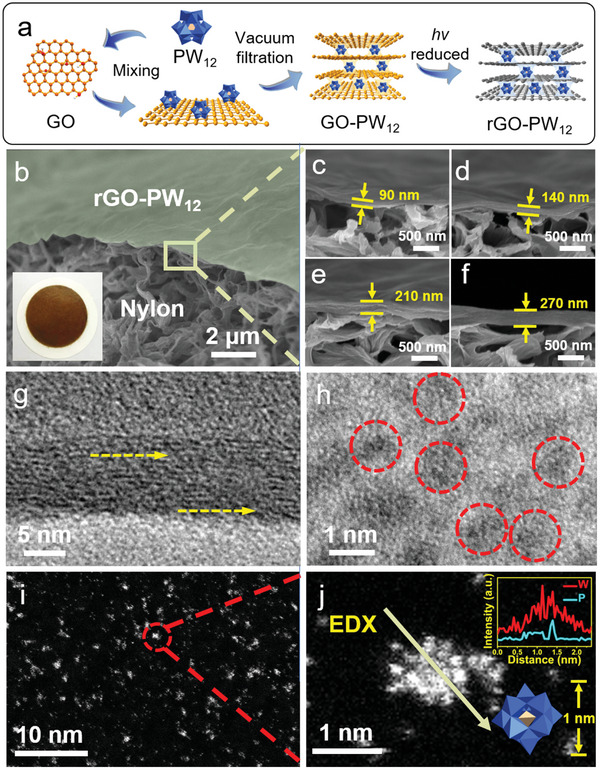
a) The scheme of the fabrication of rGO‐PW_12_ membranes through a PW_12_ assisted in situ photoreduction strategy. b) SEM image of the top view of the rGO‐PW_12_ membranes. (inset: digital photo) c–f) SEM images of the cross‐section of rGO‐PW_12_ membranes with different thicknesses. g) HRTEM image of highly ordered nanochannels of rGO‐PW_12_ membranes with one orientation. The yellow dashed arrows were eye‐guiding lines indicating the orientations of rGO. h) HRTEM image of PW_12_ cluster in rGO‐PW_12_ nanosheets. i) HAADF‐STEM image of rGO‐PW_12_ nanosheets. j) Magnified HAADF‐STEM images of PW_12_ cluster indicated by the dashed circle in Figure 1i. (inset: corresponding EDX line scanning of PW_12_ cluster and the polyhedron of PW_12_ cluster).

First of all, monolayer GO nanosheets with a thickness of ≈1 nm were obtained by exfoliation of GO prepared by modified Hummers’ method^[^ ^20^ ^]^ (Figure S1, Supporting Information). Then, the well‐mixed GO, PW_12_, and isopropanol (*i*‐Pr) solutions were deposited on nylon substrate by vacuum filtration to co‐assemble the GO‐PW_12_ membrane. As shown in **Figure** **1**a, the assembled GO‐PW_12_ membrane in the presence of *i*‐Pr was exposed directly to UV light for reduction. As a result, the PW_12_ can be photoreduced under UV irradiation accompanied by the oxidation of *i‐Pr*, and then the GO was in situ reduced to the rGO with the assistance of the photo‐reduced PW_12_.^[^ ^21^ ^]^ As shown in Figure 1b and Figure S2 (Supporting Information), scanning electron microscope (SEM) images of the rGO‐PW_12_ membrane clearly showed a smooth surface with some wrinkles of the stacked rGO‐PW_12_ nanosheets on the Nylon support. The EDX mapping images of the rGO‐PW_12_ membranes for C, O, P, and W determination proved the PW_12_ was evenly dispersed in the membrane. Representative photos of a typical rGO‐PW_12_ membrane (3.6 cm in diameter) showed a brown separation layer uniformly distributed on the substrate. The large‐area membrane (13 cm in diameter) was successfully prepared. (Figure S3, Supporting Information). The bent membrane maintained its structural integrity, indicating the membrane flexibility and good adhesion between the membrane layer and substrate (Figure S4, Supporting Information). Figure 1c–f shows the cross‐section of rGO‐PW_12_ membranes with continuous laminate structure. The thicknesses of rGO‐PW_12_ membranes can be tuned from 90 to 270 nm by adjusting the volume of rGO and PW_12_ solution on the support. The thin‐layer morphology of rGO‐PW_12_ was confirmed by transmission electron microscopy (TEM) in Figure S5 (Supporting Information). The corresponding high‐resolution Transmission Electron Microscope (HRTEM) images showed the highly ordered 2D channels of rGO laminates in Figure 1g. The high dispersion of the PW_12_ clusters on rGO nanosheets can be seen in Figure 1h. Furthermore, through the high angle annular dark field‐scanning transmission electron microscopy (HAADF‐STEM) image in Figure 1i, the bright spots can be clearly observed, which further verified the PW_12_ clusters were homogeneously dispersed on the rGO nanosheets. The magnification in Figure 1j shows PW_12_ clusters with size of 1 nm and the energy‐dispersive X‐ray spectroscopy (EDX) line scanning revealed the distributions of P and W elements in PW_12_ cluster.

**Figure 2:**
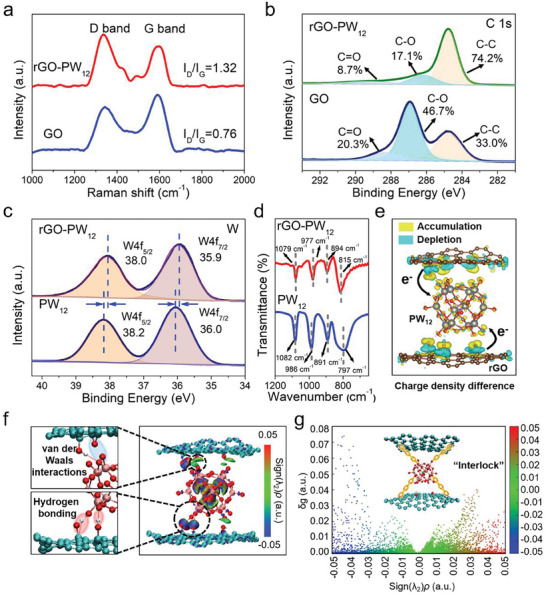
a) Raman spectra of GO and rGO‐PW_12_ membranes. b) C 1s high‐resolution XPS spectra of GO and rGO‐PW_12_ membranes. c) W high‐resolution XPS spectra of rGO‐PW_12_ membrane and PW_12_. d) FT‐IR spectra of rGO‐PW_12_ membrane and PW_12_. e) The electron density difference of rGO‐PW_12_. The yellow and blue regions indicated the charge accumulation and depletion, respectively. (isosurface value: 0.02 e Å^−3^) The curved arrows indicated the electronic transfer from rGO to PW_12_. f) Isosurface and g) scatter graph of independent gradient model (IGM) for unveiling the interaction between rGO and PW_12_, C‐cyan, H‐white, O‐red, W‐pink, P‐olive drab. (inset: the schematic diagram showed that PW_12_ was tightly bound to rGO nanosheets).

To illustrate the physical and chemical state of membranes, we carried out the following characterization. The Raman spectroscopy of the GO membrane clearly showed the characteristic D (1345 cm^−1^) and G (1595 cm^−1^) bands with a low‐intensity ratio (I_D_/I_G_∼0.76) in **Figure** **2**a, in which the D band was associated with the disorder degree from the amorphous carbon, while the G band was in connection with the in‐plane stretching vibrations of the C─C bonds the ordered sp_2_ carbons within the graphene sheets.^[^ ^22^ ^]^ The increase of I_D_/I_G_ ratio indicated the reduction of GO membrane. By comparison, the I_D_/I_G_ ratio of rGO‐PW_12_ membrane (1.32) was higher than that of rGO membrane (1.17) in Figure S6 (Supporting Information), indicating that rGO‐PW_12_ membranes possessed higher reduction degree, and the presence of PW_12_ may promote the reduction of GO membranes.

X‐ray photoelectron spectroscopy (XPS) measurements were conducted to explore the chemical environment of GO and rGO‐PW_12_. The XPS survey scan of GO membrane presented two peaks corresponding to the presence of O 1s and C 1s, while the XPS survey scan of rGO‐PW_12_ showed the signals of W, indicating the existence of PW_12_ (Figure S7, Supporting Information). Figure 2b showed the C1s peaks of the pristine GO membrane at 288.3, 286.9, and 284.8 eV, which were associated with C═O, C─O, and C─C bonds, respectively. Meanwhile, it also showed the different proportions of these groups. Compared with GO membrane, the proportion of C─C groups in rGO‐PW_12_ membrane showed a significant increase from 33.0% to 74.2%, while the C═O and C─O groups were decreased, which can be attributed to the breakup of oxygen functional groups. In addition, the reduction of GO was also evidenced by observed absorption peak shift in ultraviolet–visible (UV–vis) spectra. In UV–vis spectra (Figure S8, Supporting Information), the absorption peak of GO at ≈230 nm was red‐shifted to ≈270 nm, because of the restored electronic conjugation of rGO nanosheets after UV irradiation. The absorption band at 215 nm was ascribed to PW_12_.

As shown in Figure 2c, in the W 4f XPS spectra of rGO‐PW_12_, two signals centered at 38.0 and 38.9 eV corresponded to W 4f5/2 and W4f 7/2, indicating the presence of PW_12_ species with +6 valence state of W. The positive shift for the peak of W 4f and P 2p was observed for rGO‐PW_12_ compared with PW_12_ in Figure 2c and Figure S9 (Supporting Information). These results were attributed to the photoreduction process influencing the oxidation state of P and W elements, indicating the electronic transfer between rGO and PW_12_.^[^ ^23^ ^]^

The chemical composition of rGO‐PW_12_ was confirmed by Fourier transform infrared (FT‐IR) spectra. In Figure 2d, the rGO‐PW_12_ showed four characteristic peaks of PW_12_ at 1079, 977, 894, and 815 cm^−1^, respectively, which can be assigned to the P‐Oa, W‐Od, W‐Oc‐W, and W‐Od‐W asymmetric stretching vibrations. While those peaks in pure PW_12_ centered at 1082, 986, and 891, 797 cm^−1^, the presence and slight shift of these peaks indicated the integrity of PW_12_ and the interactions between PW_12_ and rGO.^[^ ^24^ ^]^

The charge density difference map was calculated to further investigate the electron transfer between the rGO and PW_12_. As shown in Figure 2e, the electron depletion on rGO and the electron accumulation on PW_12_ indicated the transfer of electrons from the rGO nanosheets to the PW_12_ cluster,^[^ ^25^ ^]^ which was in good agreement with the XPS and FT‐IR result.

To interpret the interaction between rGO and PW_12_, non‐covalent interaction analysis based on a molecular dynamic simulation using the independent gradient model (IGM), which method enabled the identification and visualization of regions of weak interaction. The isosurface was mapped by the different colors of the real space function *sign*(λ_2_)*ρ*. The color‐filled isosurface versus sign(*λ_2_*)*ρ* map is shown in Figure 2f and the default color transition is blue‐green‐red. The blue region in isosurface implied the strong attractive interaction such as hydrogen bonding. The green region was regarded as van der Waals interactions. The difference was that the red region signified the strong mutual exclusion interactions.^[^ ^25a^ ^]^ It can be found that there were hydrogen bonding and van der Waals interactions between rGO and PW_12_, the interaction active sites were carboxyl and hydroxyl hydrogen atoms from the surface of rGO nanosheets and the hydrogen atoms of PW_12_. The scatter graph and the isosurface sign(*λ_2_*)*ρ* mapped intermolecular electron density gradient difference for the model was shown in Figure 2g. The green to blue region indicated that the van der Waals interactions and hydrogen bonding was the dominant interaction between rGO and PW_12_ for their integration in the laminates.^[^ ^26^ ^]^ These multi‐interactions acted as chains that bound PW_12_ to the rGO nanosheets stably.

**Figure 3:**
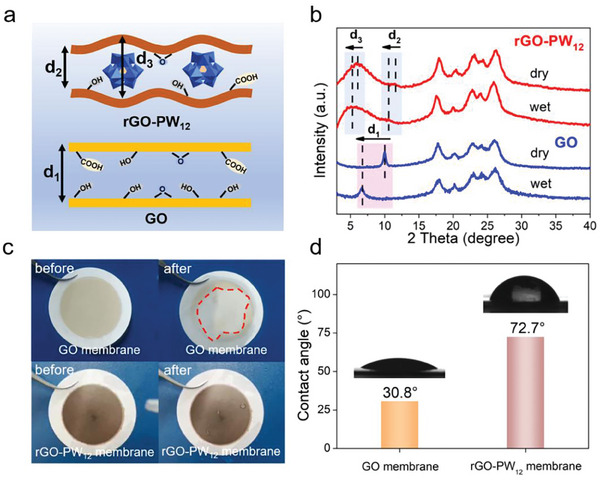
a) Schematic diagram for GO and rGO‐PW_12_ membranes structures. b) XRD patterns of pristine GO and rGO‐PW_12_ membranes in dry and wet states. c) Membrane stability test. d) The water contact angle of GO and rGO‐PW_12_ membranes.

The separation performance of the membranes was determined by the interlayer spacing.^[^ ^27^ ^]^ As shown in **Figure** **3**a, the GO membranes showed a uniform interlayer spacing; which corresponded to the interlayer distance of neighboring GO nanosheets (d_1_). The rGO‐PW_12_ membranes exhibited dual interlayer spacing, which was similar to other GO‐based membranes with dual interlayer spacing reported in the literature.^[^ ^28^ ^]^ One was the expanded interlayer spacing (d_2_), which was due to the intercalation of the PW_12_ into the neighboring rGO nanosheets. The other was the narrower interlayer distance of neighboring rGO nanosheets (d_3_).

The swelling properties of GO and rGO‐PW_12_ membranes in an aqueous environment were studied via XRD in Figure 3b. The diffraction peaks were obtained by XRD and the interlayer spacing further calculated using the Bragg's law. The peaks ranging from 17° to 26° were related to nylon substrate.^[^ ^28a^ ^]^ For GO membranes, the XRD diffraction peak at 9.90° for the dry sample shifted to 6.76° for the wet sample after being soaked in water, corresponding to the interlayer spacing of GO membranes increased from 8.92 to 13.06 Å (d_1_). In contrast, for rGO‐PW_12_ membranes, the XRD diffraction peak at 6.08° and 11.53° for dry samples shifted to 5.28° and 10.56° for wet samples after being soaked in water, respectively, corresponding to the interlayer spacing of rGO‐PW_12_ membrane increased from 14.52 to 16.41 Å (d_3_) and 7.67 to 8.37 Å (d_2_). Such a small shift for rGO‐PW_12_ membranes can be corresponded to good anti‐swelling ability of rGO‐PW_12_ membranes, which can be attributed to the strong interactions between PW_12_ and rGO and it was beneficial to improve the sieving performance of the membranes.^[^ ^29^ ^]^

Besides, the GO and rGO‐PW_12_ membranes were treated by rinsing and immersion to verify the structural stability. As shown in Figure 3c, when the rGO‐PW_12_ membrane was rinsed by water, the primitive structure can be well preserved. In contrast, a large area of GO can be peeled off from the nylon substrate immediately when it was rinsed with water. Moreover, long‐term stability was essentially important for the solution‐phase applications of the GO‐based membranes, the rGO‐PW_12_ membranes were very stable and no visible damage or delamination can be observed even after 7 days in water in Figure S10 (Supporting Information).

What's more, the surface hydrophilicity of GO and rGO‐PW_12_ membranes was determined by contact Angle analyzer. According to the water contact angle test (WCA) in Figure 3d, the contact angles were measured as 30.8° (GO membrane) and 72.7° (rGO‐PW_12_ membrane), respectively. The increase of WCA meant the removed oxygen‐containing groups of GO nanosheets, which would facilitate the anti‐swelling properties of the rGO‐PW_12_ membranes. It should be mentioned that for atomic graphene‐based membranes (WCA ≈85°), high water fluxes were considered achievable due to the rapid frictionless flow of water molecules in the interlayer capillary channels.^[^ ^30^ ^]^ Besides, the WCA of the rGO‐PW_12_ membranes with different mass ratio of rGO to PW_12_ are shown in Figure S11 (Supporting Information), implying the increase of PW_12_ led to the surface become more hydrophobic.

### 2.2 Ion Transport and Water Desalination Performance of rGO‐PW_12_ Membranes

**Figure 4:**
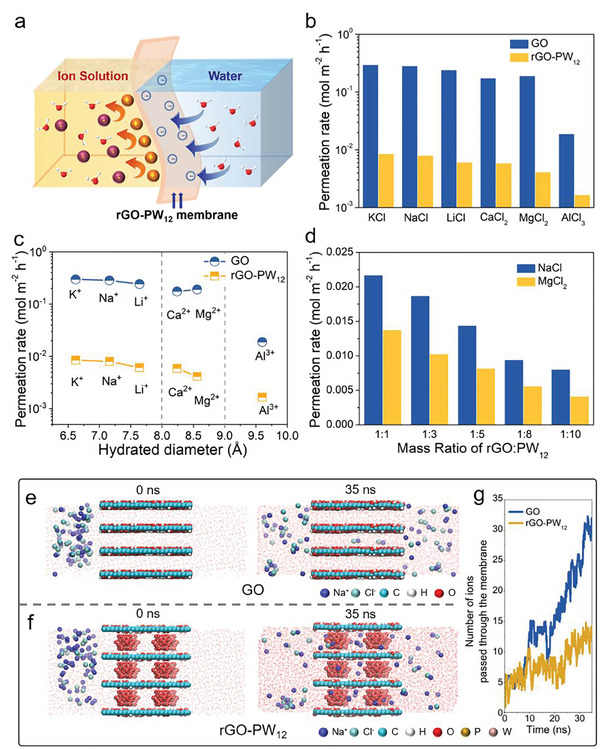
Ion permeation tests through GO and rGO‐PW_12_ membranes Feed solutions of 0.1 m KCl, NaCl, LiCl, MgCl_2_, CaCl_2_, and AlCl_3_ were used in draw side, while DI water filled the permeate side, respectively. a) Scheme of rGO‐PW_12_ membranes how to realize controllable ion transport. b,c) Comparison of the permeation rate of ions through GO and rGO‐PW_12_ membranes. d) The dependence of permeation rate on the mass ratio of rGO:PW_12_ for NaCl and MgCl_2_. MD simulation snapshots at 0 and 35 ns for two sets of Na^+^ and Cl^−^ permeation systems: untreated GO (e) and rGO‐PW_12_ (f) membranes. g) The number of ions that passed through the membranes as a function of simulation time.

The ion sieving performance of GO and rGO‐PW_12_ membranes was tested and compared toward a few common ions to evaluate the separation efficiency. The ion transport behavior driven by concentration gradient was investigated in a U‐shaped device (Figure S12, Supporting Information) with the same volume of salt solution and deionized water. Magnetic stirring method was adopted on both the feed side and the draw side to reduce the concentration polarization. The membranes we used all kept the thickness of ≈270 nm unless specified. For rGO‐PW_12_ membranes, the permeation rate of K^+^, Na^+^, Li^+^, Ca^2+^, Mg^2+^, and Al^3+^ reached 0.00851, 0.00802, 0.00611, 0.00591, 0.00413, and 0.00166 mol m^−2^h^−1^, respectively, which was similar to previous studies (**Figure** **4**a,b).^[^ ^3^, ^31^ ^]^ In comparison, the permeation rate of these ions through rGO‐PW_12_ membranes were nearly two orders of magnitude lower than through GO membranes. The relatively poor ion rejection of GO membranes was reasonable considering the effective empty channel size of 9.66 Å in wet state (the interlayer spacing of wet GO membranes was 13.06 Å, the thickness of a monolayer GO nanosheet was 3.4 Å),^[^ ^3^, ^6^, ^15^ ^]^ while after the reduction and the modification of PW_12_ cluster, the effective empty channel size of rGO‐PW_12_ membranes was narrowed down to 4.97 Å that can obstruct the passage of ions. That was, such improved ion sieving performance of rGO‐PW_12_ membranes can be attributed to the size exclusion effect and nanoconfinement effect cause that the confined interlayer spacing of the sub‐nanochannels was narrower than the hydrated diameters of these cations (Table S1, Supporting Information). Such improved ion sieving performance was superior to that of previously reported 2D membranes (Table S2, Supporting Information). Additionally, GO‐PW_12_ and rGO membranes also exhibited poorer ions rejection relative to rGO‐PW_12_ membranes (Figure S13, Supporting Information), which demonstrated that the interaction between rGO and PW_12_ greatly enhanced ion rejection performance of membranes.

As shown in Figure 4c, the rGO‐PW_12_ membranes demonstrated simultaneous improved blocking ability with the increasing mass ratio of rGO to PW_12_, showing the indispensable roles of PW_12_ site decoration in creating physically confined nanochannels for realizing efficient ion sieving. The ion transport behavior through rGO‐PW_12_ membranes with different thickness is investigated in Figure S14 (Supporting Information). As the thickness increased, the ion permeation exhibited a continuous downward trend owing to the increased transport resistance, implying the construction of well‐assembled rGO laminates was favorable for subsequent separation.^24^ We also examined how the concentration affected the Na^+^ and Mg^2+^ permeation behavior in Figure S15 (Supporting Information). With salt concentrations of 0.05 to 1.0 mol L^−1^, the Na^+^ and Mg^2+^ permeation rate also increased due to the amplification in salt concentration increased the driving force of Na^+^ and Mg^2+^ permeation.

Molecular dynamics (MD) simulations were conducted to gain insights into salt permeation and water transport in the membranes (Figure 4e–g). There were two models here, one was pristine GO membrane whose interlayer spacing in the wet state was 13.06 Å (d_1_), and the other was rGO‐PW_12_ membrane whose interlayer spacing in the wet state was 16.41 Å (d_3_). Water molecules existed both in the feed and permeate cavities. Na^+^ and Cl^−^ were placed on one side of two membranes. When the time was 0 ns, both Na^+^ and Cl^−^ were at a standstill in the feed cavities. As time went on, these ions would then permeate through interlayer channels of the membrane into the permeate cavities. When the time was 35 ns, it can be obviously viewed that several Na^+^ and Cl^−^ permeated to the other side of the GO membrane, while only a few ions can be observed in the rGO‐PW_12_ membrane, denoting a great promotion of ion rejection for the rGO‐PW_12_ membrane. As shown in Figure 4g, during the long simulation of 35 ns, the number of ions passing through the rGO‐PW_12_ membrane was much less than that of the GO membrane, which was in good agreement with the experimental results.

**Figure 5:**
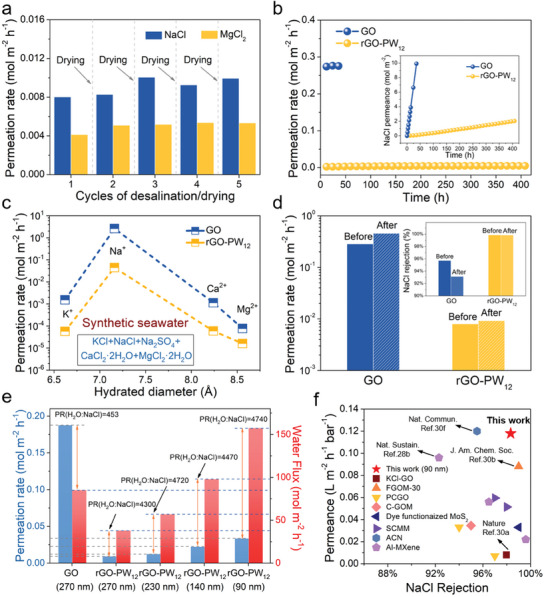
a) Cycles of desalination/drying performance of rGO‐PW_12_ membranes. b) Long‐term Na^+^ permeation rate through GO and rGO‐PW_12_ membranes. (Inset: accumulated permeated Na^+^ along with testing time.) c) Comparison of permeation rate of ions in synthetic seawater through GO and rGO‐PW_12_ membranes. The synthetic seawater was a mixture of KCl, 0.0093 m; NaCl, 0.42 m; Na_2_SO_4_, 0.029 m; CaCl_2_·2H_2_O 0.011M  and MgCl_2_·2H_2_O, 0.056 m. (Inset: Rejection rate for each cation.) d) Chlorine resistance of GO and rGO‐PW_12_ membranes. Na^+^ permeation rate and rejections (inset) through the GO and rGO‐PW_12_ membranes before and after NaClO (200 ppm) treatment for 48 h. e) Salt permeation rate and water flux of GO and rGO‐PW_12_ membranes with different thicknesses under osmotically driven pressure. f) Water permeance and NaCl rejection performance of different 2D laminar membranes using osmotically driven pressure. The detailed data are listed in Table S3 (Supporting Information).

In cyclic experiments using Na^+^ and Mg^2+^ solution, rGO‐PW_12_ membranes went through washing and drying (or drying only) after each testing session, to mimic the real membrane operating situations (**Figure** **5**a). Although a slight increase during the cycle operation mode, the permeation rate of Na^+^ and Mg^2+^was still stable at levels of 10^−3^ mol m^−2^ h^−1^. From the result of long‐term stability tests, the rGO‐PW_12_ membranes can be operated for at least 400 h with stable ion rejection performance, while the GO membranes showed an obvious upward trend in 36 h, which indicated the outstanding non‐swelling stability of rGO‐PW_12_ membranes (Figure 5b).

Then, to better simulate the real‐world ion rejection performances of rGO‐PW_12_ membranes, the mixed salt solution of synthetic seawater was used as the feed solution. As shown in Figure 5c, the permeation rate of each cation in synthetic seawater was greatly suppressed by rGO‐PW_12_ membranes compared with the GO membranes, which indicated that the blocking ability of rGO‐PW_12_ membranes on various ionic components was extremely enhanced. In addition, chlorination was widely used to prevent membrane biological contamination in water treatment applications, such as municipal water supplies and seawater desalination. However, it greatly hindered the life and performance of the membranes, so chlorine resistance was an important property for separation membranes.^[^ ^32^ ^]^ Therefore, chlorine‐resistance tests for were performed using a static approach. The GO and rGO‐PW_12_ membranes were initially immersed in NaClO solution for 48 h, then removed from the solution and thoroughly rinsed with deionized water for further ion permeation tests. It was found that the rGO‐PW_12_ membranes showed better chlorine tolerance than the GO membranes in Figure 5d. And due to the antibacterial property of PW_12_, dynamic biofouling experiments of the forward osmosis system proved that rGO‐PW_12_ membrane showed good anti‐fouling ability in Figures S16 and S17 (Supporting Information).

In order to study membranes selectivity toward water and salt, we conducted forward osmosis experiments utilizing 1 M sucrose as the draw solution and 0.1 M NaCl as the feed solution (Figure 5e). For the 270 nm‐thick rGO‐PW_12_ membranes, more than 99.5% NaCl could be rejected with a water flux of ≈97.46 mol m^−2^ h^−1^. The intercalated PW_12_ between the adjacent rGO nanosheets could not only fix the interlayer distance, but also act as pillars for fast water permeation. As expected, optimizing the membranes thickness from 270 to 90 nm led to a further increase in water flux up from 0.02829 to 0.11790 L m^−2^h^−1^bar^−1^, while maintaining high NaCl rejection of 99.5−98.3%, and displaying an ultrahigh water/salt selectivity at 4300–4740, which surpassed most the 2D nanomaterial laminar membranes (Figure 5f; Table S3, Supporting Information).^[^ ^9^, ^31^, ^33^ ^]^ Considering the ultrahigh water/salt selectivity, the ultrathin rGO‐PW_12_ membranes were deemed a suitable option for ion separation and desalination applications.

**Figure 6:**
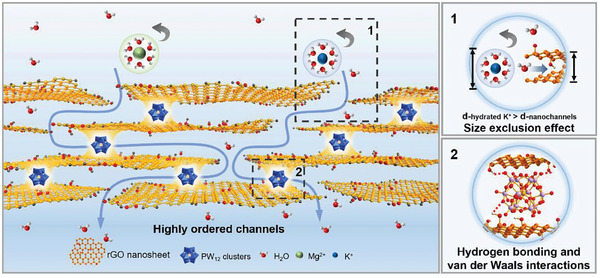
Schematic representation of efficient ion sieving (K^+^ or Mg^2+^) by the 2D rGO‐PW_12_ membranes.

In general, we systematically investigated the transport behavior of water molecular and cations in 2D sub‐nanochannels through the GO and rGO‐PW_12_ membranes by combining permeation experiments and theoretical calculations. The effects of the mass ratio of rGO to PW_12_, the thickness of the membranes and the concentration of salt solutions on the ion transport of rGO‐PW_12_ membranes were also researched. All above confirmed that the PW_12_ cluster can be used to construct stable and precise sub‐nanochannels thereby improving both the water flux and the cation rejection of rGO‐PW_12_ membranes. First, PW_12_, as a molecular cluster, can facilitate the formation of highly ordered membrane structure when intercalated into rGO nanosheets and effectively mitigate the defects between the common nanospacers and 2D membrane materials; second, the van der Waals interactions and hydrogen bonding make PW_12_ tightly attached to rGO nanosheets, which benefited the anti‐swelling and stability of rGO‐PW_12_ nanosheets; finally, the size exclusion effect and nanoconfinement effect of sub‐nanochannels achieved the high water flux and high rejection of cations (**Figure** **6**).

## 3 Conclusion

In summary, we proposed an efficient POM‐assisted in situ photoreduction strategy for fabricating rGO‐PW_12_ membranes. This facile strategy afforded a highly ordered lamellar architecture with the interlayer spacing that can be controlled at the sub‐nanometer scale by intercalating the PW_12_ cluster into rGO nanosheets. Compared with GO membranes, the permeation rate of the as‐fabricated rGO‐PW_12_ membranes for cations decreased by two orders of magnitude. What's more, the ordered transport nanochannels of the rGO‐PW_12_ membranes at sub‐nanometer scale allowed for high water flux (0.11790 L m^−2^ h^−1^ bar^−1^), NaCl rejection (98.3%) and ultrahigh water/salt selectivity of 4740. Due to the strong interactions between PW_12_ and rGO, the rGO‐PW_12_ membranes exhibited not only excellent long‐term stability up to 400 h, but also good chlorine resistance. The 2D rGO‐PW_12_ nanomembranes show great potential for the seawater desalination.

## 4 Experimental Section

Detailed experimental information is reflected in the Supporting Information.

## Conflict of Interest

The authors declare no conflict of interest.

## Supporting information

Supporting Information

## Data Availability

The data that support the findings of this study are available from the corresponding author upon reasonable request.
