# Supplementary material for: Polyoxometalate Clusters Confined in Reduced Graphene Oxide Membranes for Effective Ion Sieving and Desalination
Source: Adv Sci (Weinh). 2024 Jun 17;11(36):2402018. doi: 10.1002/advs.202402018 (PMC11422814; doi:10.1002/advs.202402018)
Supplement: Supplementary file 1 — Supporting Information [file ADVS-11-2402018-s001.pdf]

ADVANCED SCIENCE
Open Access

Supporting Information

for *Adv. Sci.*, DOI 10.1002/advs.202402018

Polyoxometalate Clusters Confined in Reduced Graphene Oxide Membranes for Effective Ion Sieving and Desalination

Yixin Yang, Wan-Lei Zhao, Yubing Liu, Qin Wang, Ziheng Song, Qinghe Zhuang, Wei Chen and Yu-Fei Song**

Supporting Information

Polyoxometalate Clusters Confined in Reduced Graphene Oxide Membranes for Effective Ion Sieving and Desalination

Yixin Yang, Wan-Lei Zhao, Yubing Liu, Qin Wang, Ziheng Song, Qinghe Zhuang, Wei Chen, and Yu-Fei Song**

Experimental Section

Materials

All chemicals used were analytical-reagent grade and received without any further purification. Graphite powder (99.95%), sulfuric acid (H_2SO_4 98%), hydrogen peroxide (H_2O_2 30%), potassium permanganate ($KMnO_4$), sodium nitrate ($NaNO_3$) were obtained from Alfa Aesar. The following saltswere used in ion permeation test experiments: potassium chloride (KCl), sodium chloride (NaCl), lithium chloride (LiCl), and aluminium chloride hexahydrate ($AlCl_3·6H_2O$) Sodium sulfate (Na_2SO_4), calcium chloride dihydrate ($CaCl_2·2H_2O$), and Magnesium chloride dihydrate ($MgCl_2·2H_2O$) were purchased from Beijing Chemical Reagent Co., Ltd. $H_3PW_{12}O_{40}$ (PW_{12}), sucrose, sodium hypochlorite and bovine serum albumin (BSA) were purchased from Energy Chemical. Nylon membranes (0.2 μm pore size, 47 mm diameter) was supplied by Tianjin JinTeng. Ultrapure water (0.0549 $μs.cm^{-1}$) was made in our laboratory.

Preparation of rGO-PW_{12} Membranes.

Graphene oxide (GO) was prepared by modified Hummer's method using natural graphite as the source [1]. To obtain the single-layer GO, the GO powder was dissolved in ultrapure water and diluted to 0.1 $mg·mL^{-1}$, then the solution was subjected to bath-sonication for 1 hour and ready for use. PW_{12} was dissolved in ultrapure water and diluted to 1 $mg·mL^{-1}$. Isopropanol, GO solution and PW_{12} solution was mixed in a certain proportion and ultrasound 0.5 h before preparing the membrane. After vacuum-assisted filtration of the mixed solution on nylon substrate, a laminar GO-PW_{12} layer was formed. Then, the prepared GO-PW_{12} membrane was exposed with UV light for *in-situ* photo reduction. After UV irradiation for 4 hours, the synthesized rGO-PW_{12} membrane was obtained. In preparing the membrane, rGO-PW_{12} membranes with different mass ratio of rGO to PW_{12} and thickness were obtained by controlling the volume of GO and PW_{12} solution.

Characterizations

The detail, height profiles and roughness of the membrane surface were measured by atomic force microscopy (AFM, Multimode Nanoscope IIIa system, Veeco Instruments). The morphologies and thicknesses of the membranes were obtained by Scanning electron microscopy (SEM, Zeiss Supra 55 VP) with energy dispersive X-ray (EDX) analysis of C, O, P and W elements. Transmission electron Microscopy (TEM) images were conducted on a JEOL JEM-2010 electron microscope operating at 200 kV to analyze the morphology of as-prepared GO-PW_{12} sheets. High resolution TEM (HR-TEM) was performed on JEOL JEM-2100 under an accelerating voltage of 400 kV. HAADF-STEM measurements were obtained using a JEOL-2100FS and an Oxford Xmax 80T instrument. For creation of the size histograms, the diameters of more than 100 particles were measured, fitted by log normal distribution functions. Fourier transform infrared spectroscopy (FT-IR) spectra were recorded on Bruker Vector 22 infrared spectrometer. Raman spectroscopy (Renishaw Raman spectrometer) was carried out with 580 nm wavelength incident laser light in the range of 1000 to 2000 cm^{-1}. We utilized X-ray photoelectron spectroscopy (monochromatized Al Kα exciting X-radiation PHI Quantera SXM) to investigate the chemical structure of the membranes. X-ray diffraction (XRD) analysis was carried out on Rigaku XRD-6000 diffractometer equipped with a Cu Ka radiation (λ = 1.5405 Å) to characterize the structure of prepared membranes and further calculate the interlayer spacing of the membranes. The UV-Vis spectrophotometer (DRS, Beijing PGENERAL, TU-1901) for molecular absorption quantitative was used to analyze the reduction of GO and the separation performances of the membranes. Water contact angles were tested by a JC2000D2M static contact angle goniometer (POWEREACH®). The Zeta potentials of GO, PW_{12}, rGO-PW_{12} solutions were obtained by a Malvern Zeta Potential Analyzer (Malvern ZS90).

Performance of rGO-PW_{12} Membranes.

Ion Permeation Measurement through the Membrane.

Ion permeation experiments were performed with a U-shaped permeability apparatus and the membrane was sealed in the middle of two compartments, for permeate side of the U-shaped permeability cell was filled with distilled water and the feed side contained various salt solution (KCl, NaCl, LiCl, $CaCl_2 \cdot 2H_2O$, $MgCl_2 \cdot 2H_2O$,

$AlCl_3 \cdot 6H_2O$) with the concentration of 0.1 M. The effective membrane area was 1.77 cm^2 (a circular hole with a radius of 0.75 cm), and all experiments were performed at room temperature. What's more, to avoid concentration polarization during the test, both compartments were under magnetic stirring to make sure the solution was homogeneous. The conductivity meter was used to measure the conductivity of the permeate side, and the ion permeation rate as a function of permeation time was further calculated. The detection limit of the conductivity meter is 0.01 μS, corresponding to the concentration of salt at the μM level. In addition, inductively coupled plasma spectrometry (ICP) were applied to quantitatively analyze the cation species in the permeation compartment.

Desalination Performance Test.

The water flux value was also obtained using the U-shaped device, where the feed side was 50 mL with 0.1 M NaCl and the draw side was 50 mL with 1 M sucrose. The water flux (Jw) was derived from the liquid volume difference between feed side and draw side (ΔV) over time (Δt) as follows:

$$J_w = \frac{\Delta V}{A \times \Delta t}$$

According to the Van't Hoff equation, 1 M sucrose can produce osmotic pressure gradient of about 24.46 bar at room temperature. Thus, the water flux (L h^{-1} m^{-2}) obtained from the above equation can be transformed to water permeance (L h^{-1} m^{-2} bar^{-1}).

The salt rejection (R) was calculated by measuring the permeated ions in the permeate side, which can be calculated as follows:

$$R = \left(1 - \frac{C_d}{C_f}\right) \times 100\%$$

where C_d and C_f are the NaCl concentrations in the draw side and feed side, respectively.

Antifouling performance test.

The antifouling property of membranes were conducted with the feed side containing 200 ppm BSA. Firstly, the membrane was placed in the membrane cell with 10 mM NaCl in feed side and 2M NaCl in draw side. The baseline test was conducted for 360

min to obtain the initial flux (J_0) of the membrane without the addition of any foulants. The foulants were then added to the feed side and the filtration tests were run for 360 min to measure the fouling propensity of the developed membranes towards foulants. The relative flux (J_1) was to characterize the contribution of membrane fouling. After that, the membrane was cleaned with DI water for 120 min under magnetic stirring. Last, the feed side was replaced with 10 mM NaCl and the draw side was replaced with a 2M NaCl to observe the recovery of water flux (J_2). The relative flux was used to measure the flux reduction before cleaning as a means of comparing the flux reduction before cleaning as a means of comparing the flux changes before and after cleaning. The flux recovery rate (F_{rr}) was calculated using Eq. (1):

$$Frr = \left(\frac{J_2}{J_0}\right) \times 100\% \tag{1}$$

The rate of total fouling (R_t) was obtained from Eq. (2):

$$R_t = \left(1 - \frac{J_1}{J_0}\right) \times 100\% \tag{2}$$

The rate of irreversible and reversible fouling was computed using the following equation (Eq. (3), (4)):

$$R_{ir} = \left(1 - \frac{J_2}{J_0}\right) \times 100\% \tag{3}$$

$$R_r = \left(\frac{J_2 - J_1}{J_0}\right) \times 100\% \tag{4}$$

MD simulations

To investigate the ion transport properties, first-principles-based MD simulations with two models were carried out using CP2K code [2]: GO and rGO-PW_{12}. The AIMD calculations was used to calculate the differential charge and the binding energy. The force and velocity are calculated using density functional theory (DFT) for Perdew – Burke–Ernzerhof (PBE) functional, Gaussian-type basis set6 and Goedecker-Teter-Hutter (GTH) pseudopotentials combined with Gamma k-point and 350 Ry plane wave cut-off [3]. The atomic models were analyzed and produced using VMD19 software. The generalized gradient approximation (GGA) with the Perdew-Burke-Ernzerhof (PBE) function was used for the evaluation of the exchange-correlation energy. The DFT-D3

dispersion correction method was used to describe the weak interaction between slabs and active species [4].

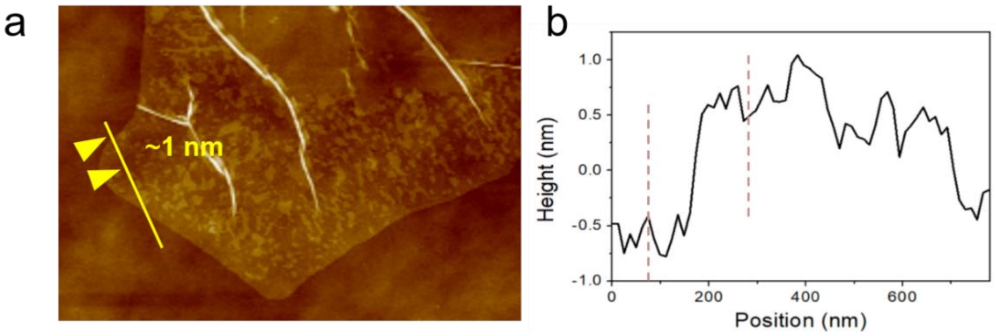

Figure S1. AFM image (a) and the height profile (b) of GO nanosheet.

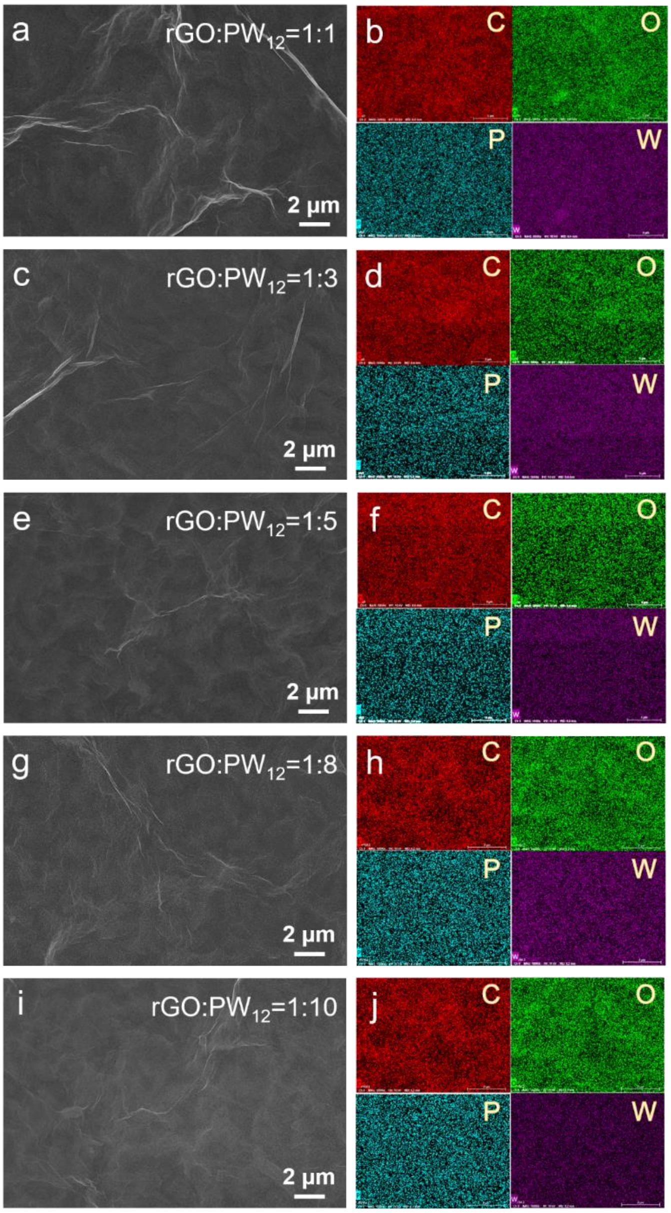

Figure S2. SEM image of the top view of the rGO-PW_{12} membrane with different mass ratio of rGO to PW_{12} and corresponding EDX mapping images.

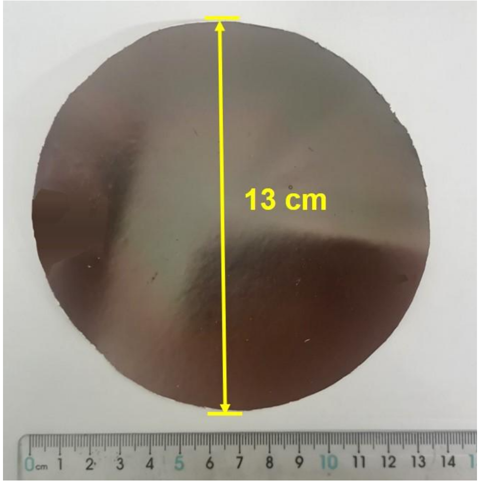

Figure S3. The digital photo of large-area rGO-PW_{12} membrane with a diameter of 13 cm (effective area: ~132 cm^2).

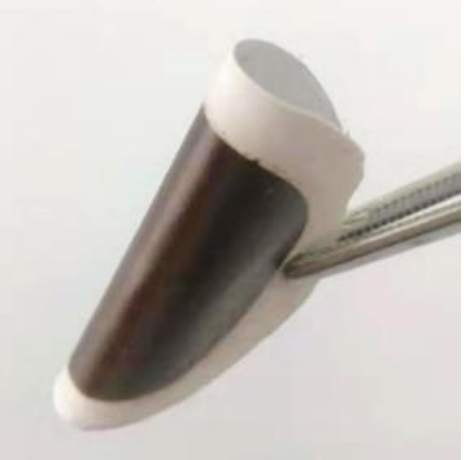

Figure S4. The optical images of bend rGO-PW_{12} membrane.

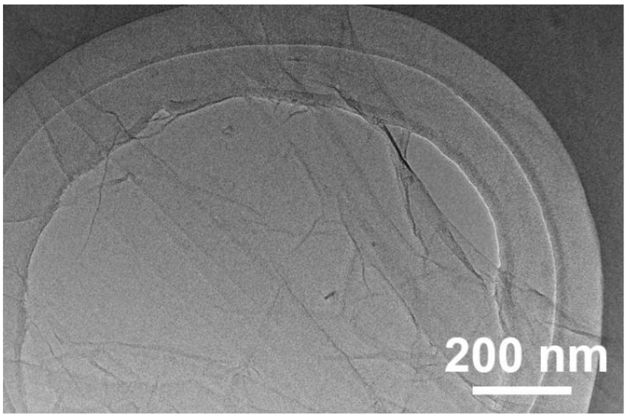

Figure S5. TEM image of rGO-PW_{12} nanosheets.

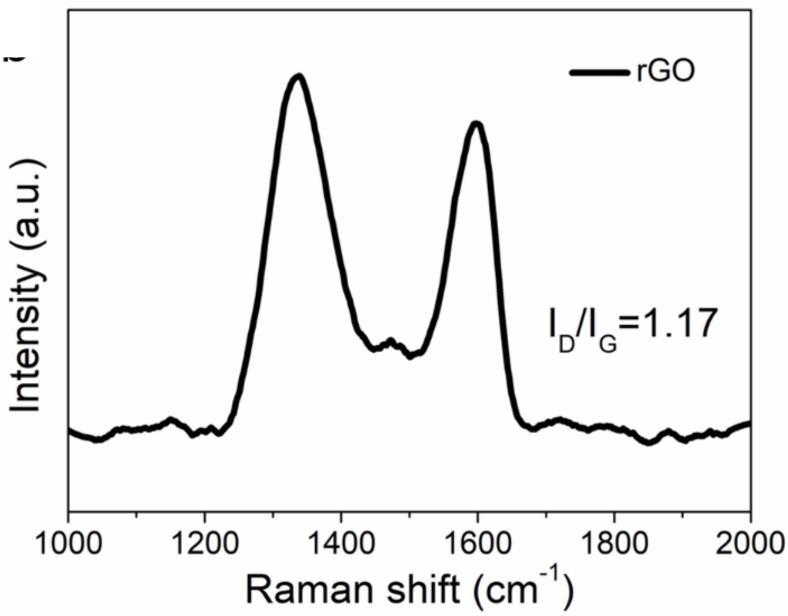

Figure S6. The Raman spectrum of rGO membrane.

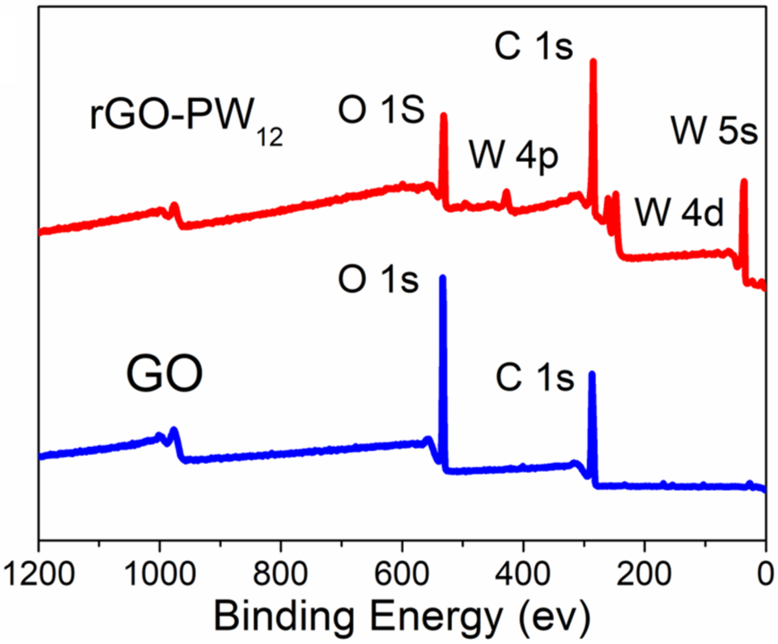

Figure S7. XPS survey scan of GO membrane and rGO-PW_{12} membrane.

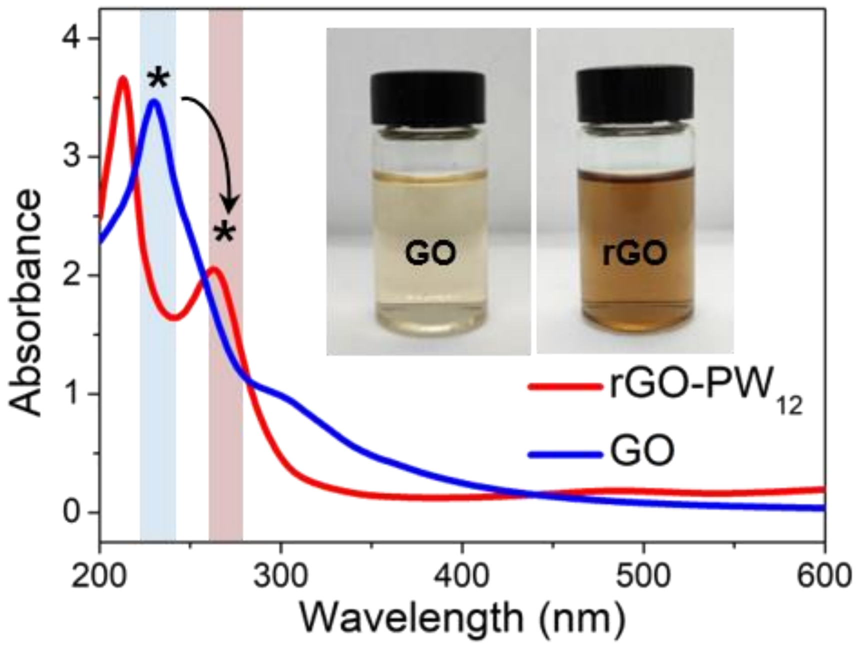

Figure S8. UV–vis absorption spectra of GO and rGO-PW$_{12}$ aqueous suspension (inset is the optical images of GO and rGO-PW$_{12}$ aqueous suspension).

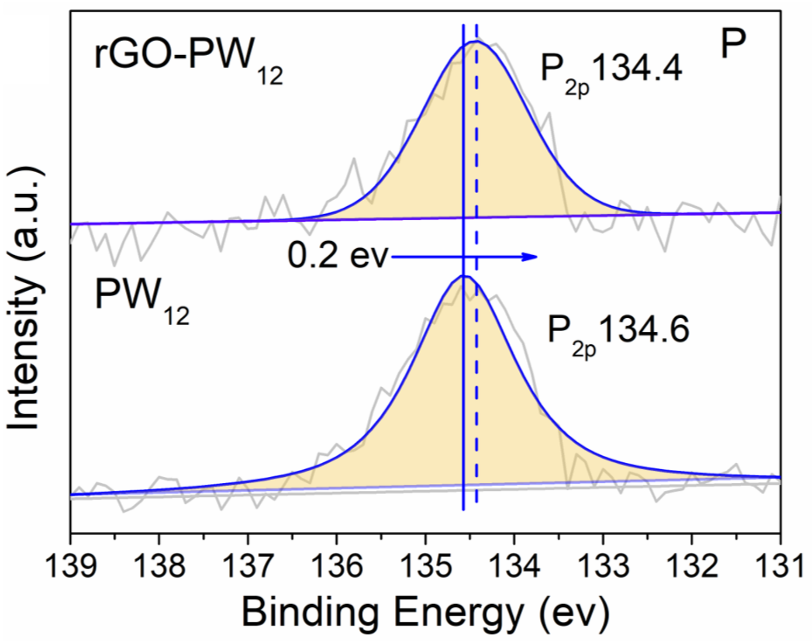

Figure S9. P high-resolution XPS spectra of rGO-PW$_{12}$ membrane and PW$_{12}$.

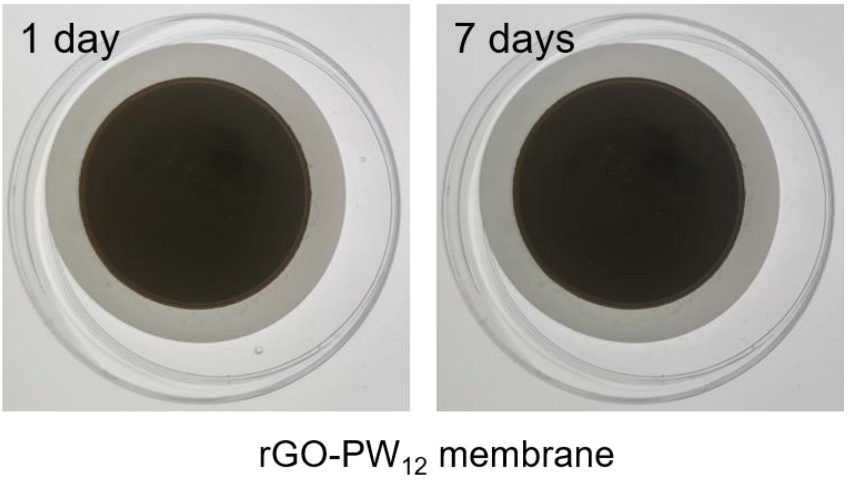

Figure S10. Photos of rGO-PW_{12} membranes were taken after the membranes were stored in water for 7 days.

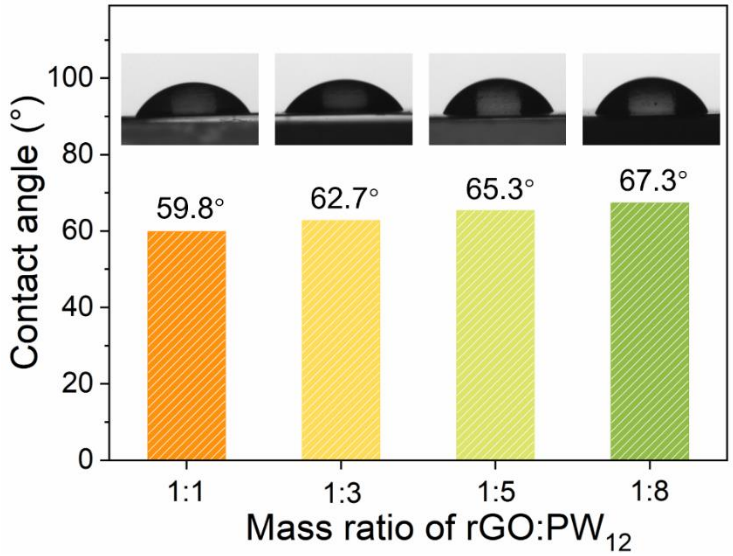

Figure S11. The water contact angle of rGO-PW_{12} membranes with different mass ratio of rGO to PW_{12}.

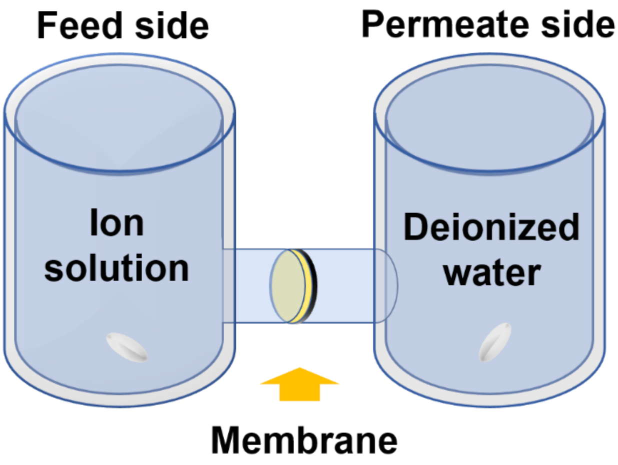

Figure S12. The H-type permeation device for ion permeation experiments.

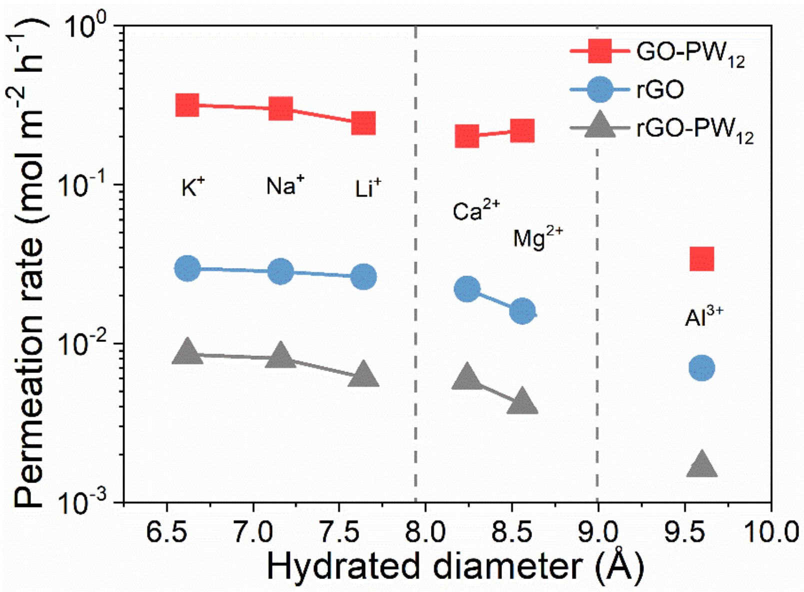

Figure S13. Comparison of the permeation rate of ions through rGO, GO-PW_{12} and rGO-PW_{12} membranes.

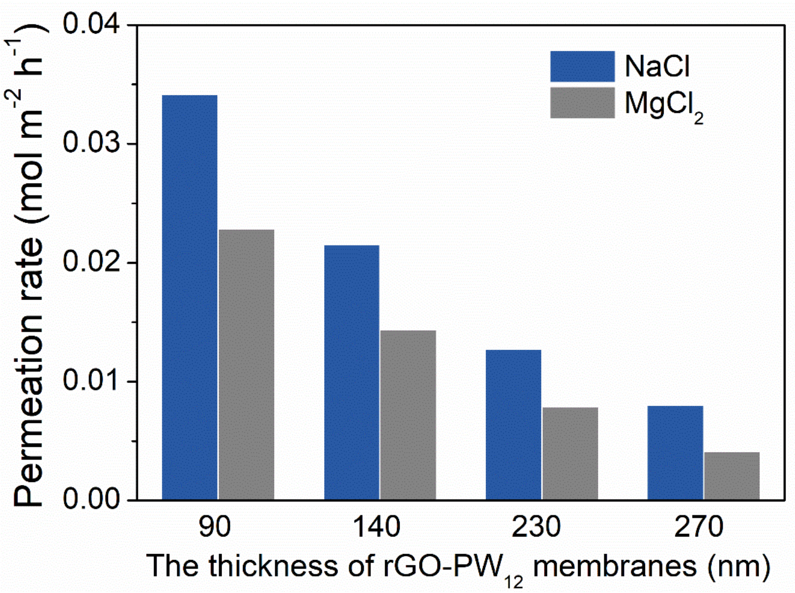

Figure S14. Effect of membrane thickness on NaCl and $MgCl_2$ permeation behaviour.

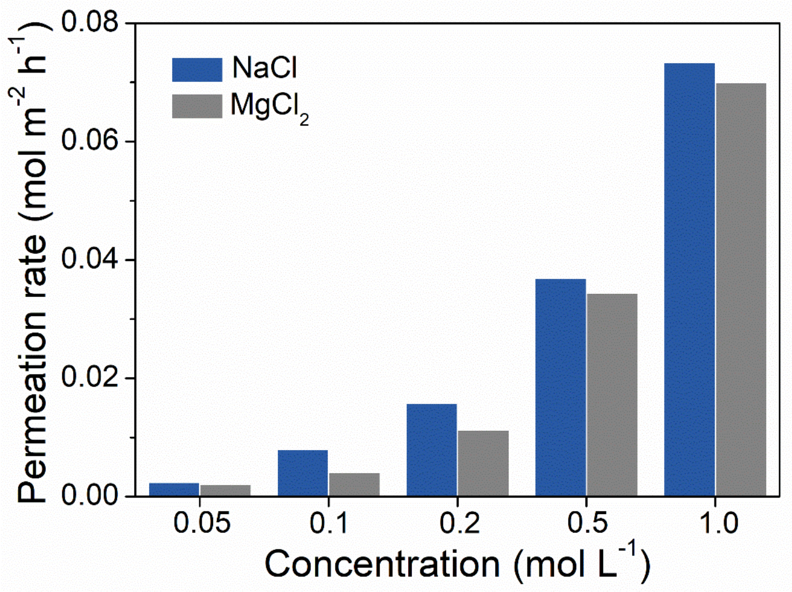

Figure S15. Effect of salt concentration on NaCl and $MgCl_2$ permeation behavior of rGO-PW_{12} membranes.

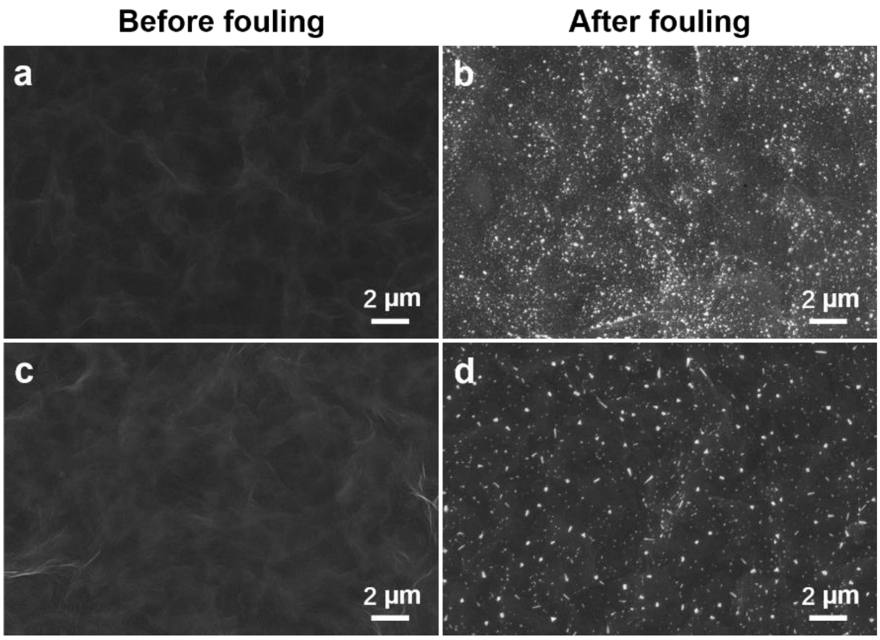

Figure S16. Original top-surface SEM images of GO (a) and rGO-PW_{12} (c) membrane before fouling. Top-surface SEM images of GO (b) and rGO-PW_{12} (d) membrane when operated FO system with feed solutions of BSA.

The rGO-PW_{12} membrane exhibited better antifouling properties than GO membrane, which relatively few pollutants attached to the surface of rGO-PW_{12} membrane can be observed.

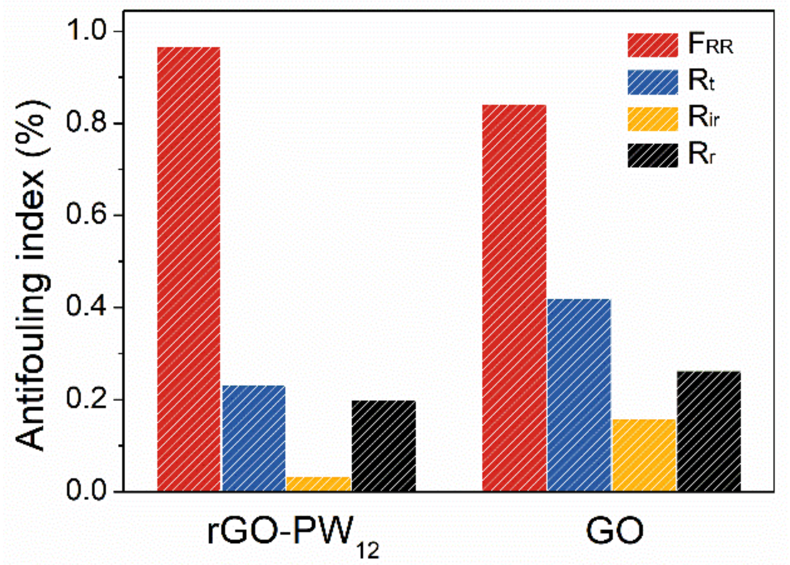

Figure S17. Fouling data of GO and rGO-PW_{12} membrane.

The dynamic experiment also proved that rGO-PW_{12} membrane can inhibit fouling, which showed higher water flux recovery rate (Frr=96.7%) and lower total fouling rate (Rt=23.3%) than GO membrane in Figure R2.

Table S1. Diameters of effective and hydrated ions used in this work.

Ions	Effective ionic diameter (Å)	Hydrated diameter (Å)
**K^{+}**	2.76	6.62
**Na^{+}**	2.04	7.16
**Li^{+}**	1.52	7.64
**Ca^{2+}**	2.00	8.24
**Mg^{2+}**	1.44	8.56
**Al^{3+}**	1.07	9.50

Table S2. Modification efficacy comparison of rGO-PW_{12} membranes with recently reported membranes based on their ion sieving performance.

Membrane Materials	Thickness (nm)	$C_{(ion\ feed)}$ (mol L^{-1})	Ion PR (mol m^{-2} h^{-1}) Before/After Treatment	Enhancement Factor	Ref.
**rGO-PW_{12}**	270	0.1 (Na^+)	0.285/0.008	35.6	This work
**GO (-NH_2&-N)**	200	0.2 (K^+)	~0.230/~0.055	4.2	[5]
**GO (expoxy confinement)**	>1000	1.0 (Na^+)	~0.005/~0.0001	29.4	[6]
**GO (EDA) Ethylenediamine**	~2200	0.1 (Na^+)	~0.113/~0.065	1.7	[7]
**GO (PPD) p-Phenylenediamine**			~0.113/~0.013	8.7	
**GO (EDA) o-Phenylenediamine**			~0.113/0.008	14.1	
**$Ti_3C_2T_x$ (EtOH)**	300	0.2 (Na^+)	0.368/0.016	23.3	[8]
**$Ti_3C_2T_x$ (Al^{3+}-O)**	1100	0.2 (Na^+)	~0.112/~0.005	25	[9]
**$Ti_3C_2T_x$ (Al_{13}-O)**	5000	0.5 (Na^+)	~0.048/~0.012	4	[10]
**$Ti_3C_2T_x$ (Ti-O-T)**	500	0.2 (Na^+)	0.222/0.007	33	[11]
**$Ti_3C_2T_x$ (Ti-O-Ti & -SO_3H)**	2000	0.2 (Li^+)	0.283/0.080	3.5	[12]

Table S3. The comparison of 2D nanomaterial membranes for desalination performance in a diffusion cell.

Types	Membrane Materials	Thickness (nm)	Water Permeance (L m^{-2} h^{-1} bar^{-1})	NaCl Rejection (%)	Ref.
**Reduced graphene oxide (rGO)**	**rGO-PW$_{12}$**	270	0.0283	99.5	This work
		230	0.0424	99.3	
		140	0.0589	98.9	
		90	0.1179	98.3	
**Graphene oxide (GO)**	**KCl-GO**	280	0.0300	n/a	[13]
		550	0.0192	n/a	
		750	0.0083	99.0	
	**FGOM-30**	200	0.0302	99.5	[5]
		100	0.0501	99.2	
		50	0.0881	99.0	
	**PCGO**	5000	0.0070	97.0	[6]
		1000	0.0330	94.0	
	**Non-covalent functionalized GOM**	5300	0.0346	95.0	[14]
**MoS$_2$**	**Dye functionalized MoS$_2$**	5000	0.0330	99.0	[15]
**MXene**	**SCCM-80**	500	0.0595	97.0	[11]
	**SCCM-120**		0.0513	98.0	
	**SCCM-180**		0.0515	98.0	
	**Al- MXene**	2700<br>1100<br>580	0.022<br>0.056<br>0.096	99.6<br>96.5<br>89.5	[16]
**Carbon nitrides (CN)**	**ACN**	320	0.1200	95.5	[17]

References

[1] F. Liang, Q. Liu, J. Zhao, K. Guan, Y. Mao, G. Liu, X. Gu, W. Jin, *AIChE J.* **2019**, *66*.

[2] J. Hutter, M. Iannuzzi, F. Schiffmann, J. VandeVondele, *Wires. Comput. Mol. Sci.* **2014**, *4*, 15-25.

[3] J. P. Perdew, K. Burke, M. Ernzerhof, *Phys. Rev. Lett.* **1998**, *77*, 3865-3868.

[4] L. Goerigk, *A Comprehensive Overview of the DFT-D3 London-Dispersion Correction*, **2017**.

[5] Y. Qian, J. Shang, D. Liu, G. Yang, X. Wang, C. Chen, L. Kou, W. Lei, *J. Am. Chem. Soc.* **2021**, *143*, 5080-5090.

[6] a) J. Abraham, K. S. Vasu, C. D. Williams, K. Gopinadhan, Y. Su, C. T. Cherian, J. Dix, E. Prestat, S. J. Haigh, I. V. Grigorieva, P. Carbone, A. K. Geim, R. R. Nair, *Nat. Nanotechnol.* **2017**, *12*, 546-550. b) Z. Jia , W. Shi , *Carbon* **2016**, *101*, 290-295; c) Z. Lu , Y. Wu , L. Ding , Y. Wei , H. Wang , *Angew. Chem., Int. Ed.* **2021**, *133*, 22439-22443.

[7] Z. Jia, W. Shi, *Carbon* **2016**, *101*, 290-295.

[8] Y. Kang, T. Hu, Y. Wang, K. He, Z. Wang, Y. Hora, W. Zhao, R. Xu, Y. Chen, Z. Xie, H. Wang, Q. Gu, X. Zhang, *Nat. Commun.* **2023**, *14*, 4075.

[9] L. Ding, L. Li, Y. Liu, Y. Wu, Z. Lu, J. Deng, Y. Wei, J. Caro, H. Wang, *Nat. Sustain.* **2020**, *3*, 296-302.

[10] J. Zhu, L. Wang, J. Wang, F. Wang, M. Tian, S. Zheng, N. Shao, L. Wang, M. He, *ACS Nano* **2020**, *14*, 15306-15316.

[11] Z. Lu, Y. Wei, J. Deng, L. Ding, Z.-K. Li, H. Wang, *ACS Nano* **2019**, *13*, 10535-10544.

[12] Z. Lu, Y. Wu, L. Ding, Y. Wei, H. Wang, *Angew. Chem. Int. Ed.* **2021**, *60*, 22265-22269.

[13] L. Chen, G. Shi, J. Shen, B. Peng, B. Zhang, Y. Wang, F. Bian, J. Wang, D. Li, Z. Qian, G. Xu, G. Liu, J. Zeng, L. Zhang, Y. Yang, G. Zhou, M. Wu, W. Jin, J. Li, H. Fang, *Nature* **2017**, *550*, 415-418;

[14] J. Ran, C. Chu, T. Pan, L. Ding, P. Cui, C.-F. Fu, C.-L. Zhang, T. Xu, *J. Mater. Chem. A* **2019**, *7*, 8085-8091.

[15] W. Hirunpinyopas, E. Prestat, S. D. Worrall, S. J. Haigh, R. A. W. Dryfe, M. A. Bissett, *ACS Nano* **2017**, *11*, 11082-11090.

[16] L. Ding, L. Li, Y. Liu, Y. Wu, Z. Lu, J. Deng, Y. Wei, J. Caro, H. Wang, *Nat. Sustain.* **2020**, *3*, 296-302.

[17] Y. Wang, T. Lian, N. V. Tarakina, J. Yuan, M. Antonietti, *Nat. Commun.* **2022**, *13*, 7339.
